# Pressure-Dependent Tuning of Photoluminescence and Size Distribution of Carbon Nanodots for Theranostic Anticancer Applications

**DOI:** 10.3390/ma13214899

**Published:** 2020-10-31

**Authors:** Nicolò Mauro, Mara Andrea Utzeri, Gianpiero Buscarino, Alice Sciortino, Fabrizio Messina, Gennara Cavallaro, Gaetano Giammona

**Affiliations:** 1 Laboratory of Biocompatible Polymers, Department of Biological, Chemical and Pharmaceutical Sciences and Technologies, University of Palermo, via Archirafi 32, 90123 Palermo, Italy; maraandrea.utzeri@unipa.it (M.A.U.); gennara.cavallaro@unipa.it (G.C.); gaetano.giammona@unipa.it (G.G.); 2 Fondazione Umberto Veronesi, Piazza Velasca 5, 20122 Milano, Italy; 3 Department of Physics and Chemistry “E. Segrè”, University of Palermo, via Archirafi 36, 90123 Palermo, Italy; gianpiero.buscarino@unipa.it (G.B.); alice.sciortino02@unipa.it (A.S.); fabrizio.messina@unipa.it (F.M.); 4 Institute of Biophysics at Palermo, Italian National Research Council, Via Ugo La Malfa 153, 90146 Palermo, Italy

**Keywords:** carbon nanodots, phototherapy, cancer therapy, theranostics, MDA-MB-231

## Abstract

Carbon nanodots (CDs) have recently attracted attention in the field of nanomedicine because of the biocompatibility, cost-effective nature, high specific surface, good near infrared (NIR) photothermal conversion into heat and tunable fluorescence properties, which have paved the way toward incorporating use of CDs into innovative anticancer theranostic platforms. However, a reliable synthesis of CDs with established and controlled physiochemical proprieties is precluded owing to the lack of full manipulation of thermodynamic parameters during the synthesis, thus limiting their use in real world medical applications. Herein, we developed a robust solvothermal protocol which allow fine controlling of temperature and pressure in order to obtain CDs with tunable properties. We obtained different CDs by modulating the operating pressure (from 8 to 18.5 bar) during the solvothermal decomposition of urea and citric acid in N,N-dimethylformamide at fixed composition. Atomic force microscopy (AFM), Fourier transform infrared (FTIR), ultraviolet-visible (UV-vis) and fluorescence spectroscopy were used to assess the role of pressure in influencing size, optical and surface properties of the obtained CDs. While preliminary biological and anticancer performance of CDs was established on the MDA-MB-231 cell line, used as triple negative breast cancer model. Our results indicate that pressure impinge on the formation of carbon nanoparticles under solvothermal conditions and impart desired optical, size distribution, surface functionalization and anticancer properties in a facile way. However, we have highlighted that a strategic surface engineering of these CDs is needed to limit the adsorption of corona proteins and also to increase the average surface diameter, avoiding a rapid renal clearance and improving their therapeutic efficacy in vivo.

## 1. Introduction

Nitrogen-rich carbon nanodots (CDs) are quasi spherical carbon nanoparticles, with size <10 nm, doped with nitrogen and functionalized on the surface with a variety of groups such as amine, amide, hydroxyl and carboxylic acid functions which allow further engineering for a variety of research and commercial applications [1,2,3,4,5]. In particular, CDs have emerged in the field of nanomedicine because of the biocompatibility, cost-effective nature, high specific surface, good near infrared (NIR) photothermal conversion into heat and tunable fluorescence properties, which have paved the way toward incorporating use of CDs into innovative anticancer theranostic architectures [1,6,7]. In fact, they have been proposed as multimodal smart platforms simultaneously providing drug delivery, selective NIR-triggered photothermal ablation of cancer cells and self-tracking fluorescence properties useful in image-guided photo-chemotherapy (IG-PCT) [5,8]. Many nanomaterials with suitable toxicity profiles, contrast features and high photothermal conversion property within the biological transparency window (620–1100 nm) have been proposed for IG-PTT applications such as carbon nanomaterials, organic dyes and noble metal nanoparticles. However, despite the high photostability of noble metal nanoparticles (e.g., gold nanorods and silver nanoparticles), they are characterized by a low renal excretion and organ accumulation, which imply potential toxicity phenomena thus limiting their clinical application. By contrast, having a diameter below the renal cut off (<5 nm), CDs can be easily eliminated avoiding off-target organ accumulation. 

Unlike other carbon nanomaterials proposed in theranostics (such as graphene oxide and carbon nanotubes) CDs can be prepared using versatile and scalable solvothermal bottom-up approaches which have shown the appeal of CDs in real-world applications [9,10,11,12] For example, cheap small molecules such as urea and citric acid can be used to prepare CDs with excellent fluorescence properties, in quantities up to Kg scales [1]. However, the huge access to starting monomers and protocols, as well as the lack of full control in all parameters involved in solvothermal synthesis of CDs such as the operating pressure, may lead to confusing interpretation of literature data and unreliable synthetic procedures. Also in microwave assisted synthesis of CDs, where operating pressure and temperature can be roughly controlled by the power output, synthetic procedures appear hard to be proposed in large-scale synthesis due to difficulties in reproducing temperature (superheating usually occurs) and pressure evolution over time [13]. In fact, since the exact power output is very difficult to regulate, temperature and pressure control are very challenging resulting in greatly varying outcomes [14]. These problems should be overcome by developing robust solvothermal protocols which allow fine controlling of temperature and pressure in order to obtain CDs with tunable size distribution, fluorescence and surface functionalization. Besides, although pressure is a thermodynamic parameter that play a crucial role in solvothermal reactions (viscosity, density and crystal lattice energy usually change with pressure), to the best of our knowledge there are not studies which report the effect of the synthesis pressure on the physicochemical properties of CDs [15]. There is no information on whether the control of pressure can be employed to control structure, size and optical properties of CDs via a simple and scalable way. 

Here, we assessed the possibility of using the operating pressure to obtain CDs with tunable properties by a bottom-up solvothermal synthesis using the urea/citric acid pathway. Attempts were made to understand what is the impact of the operating pressures on the red emission band of CDs, since red fluorescence is highly desired in fluorescence imaging and theranostic applications. It is a commonplace that red-to-NIR fluorescence is required to give detailed information from deep tissues such as tumors and thus red-emitting nanomaterials are usually needed in cancer theranostic [16,17].

Apart from fluorescence, nanoparticles which are intentionally engineered for anticancer theranostic applications require narrow and suitable size distribution and optimized surface functionalization. These parameters are involved in a variety of phenomena at the human tissue/biomaterial interface which strongly influence the fate of nanomedicines after their administration (such as plasma protein interaction, biodistribution, tissue accumulation, aggregation, etc.) [18,19,20,21]. Hence, tuning these features by simple methods is of huge interest to design next generation of CDs endowed with a tailor-made combination of properties for targeted theranostic therapies. Here, we have managed to exploit the operating pressure in order to not only tailor the main properties of nitrogen-rich CDs to exhibit different fluorescence (from green to red), NIR photothermal conversion, size distribution and surface functional groups but also to have tunable biological properties (i.e., cytocompatibility and on demand photothermal cytotoxic properties) exploitable in precision anticancer medicine. The effect of pressure on the fluorescence self-tracking and NIR photothermal ablation capabilities of CDs, obtained at different operating pressures, were studied on a model of breast cancer using the MDA-MB-231 cell line.

## 2. Materials and Methods 

Urea (99%), citric acid (99.5%), N,N-dimethylformamide (DMF), Sephadex^®^ G-15 and G-25, ethyl acetate (Chromasolv), dialysis tubing MWCO 2 kDa were purchased from Sigma Aldrich (Milan, Italy) and used as received. MDA-MB-231 cell lines was purchased from Sigma Aldrich (Milan, Italy) and cultured in supplemented Dulbecco’s Minimum Essential Medium (DMEM) (EuroClone, Milan, Italy) supplemented with 10% fetal bovine serum (FBS, EuroClone, Milan, Italy) 1% of penicillin/streptomycin (10,000 U mL^−1^ and 10 mg mL^−1^ respectively, Euroclone, Milan, Italy) and 1% of L-glutamine (EuroClone, Milan, Italy), at 37 °C in 5% CO_2_ humidified atmosphere. Cell Titer 96 Aqueous One Solution Cell Proliferation assay (MTS solution) were purchased from Promega (Madison, WI, USA).

### 2.1. Synthesis of Carbon Nanodots

Carbon Nanodots were prepared from urea (20 g) and citric acid (10 g) in DMF (100 mL) in solvothermal conditions at 160 °C for 4 h. The synthesis was replicated at three different pressure conditions, 8 bar, 13 bar and 18.5 bar, thanks to the use of an autoclave (Büchi AG, Miniclave steel type 3, Gschwaderstrasse, Switzerland) that allows us to control the reaction pressure. At different pressure conditions three different CDs were obtained, named CDs_18.5bar_, CDs_13bar_ and CDs_8bar_. The products were treated in the same way. The reaction was cooled down to r.t. and the product was precipitated dropwise in ethyl acetate (300 mL) under stirring. The precipitate was retrieved by centrifuging at 10,000 rmp and 25 °C for 15 min. The powder was dispersed in water by sonicating (15 min × 3) and filtered on 0.2 μm membrane filter by Buckner funnel. Successively, in a first step the CDs_18.5bar_ and CDs_13bar_ were purified by gel permeation chromatography using Sephadex G-25. Then, the fractions of CDs_18.5bar_, CDs_13bar_ and CDs_8bar,_ showing a red fluorescence were selected and unified, respectively. In a second step, crude CDs_8bar_ and the selected fractions of CDs_18.5bar_ and CDs_13bar_ were purified by size exclusion chromatography (SEC) using a glass column (100 cm length, 1.5 cm diameter) packed in turn with Sephadex G-25 and G-15 (Sigma Aldrich, Milan, Italy). Fractions collected for each sample were combined according to the optical properties and subsequently freeze-dried.

### 2.2. Chemical and Physicochemical Characterization of the Carbon Nanodots

The size distributions of the CDs_18.5bar_, CDs_13bar_ and CDs_8bar_ were assessed by atomic force microscopy (AFM). Samples were deposited on a mica substrate and then dried in vacuum. The measurements were performed in air with a Bruker FAST-SCAN microscope working in soft tapping mode using a FAST SCAN A probe (Billerica, MA, USA) having triangular shape, nominal tip radius of 5 nm and nominal spring constant of 17 N/m. The tip velocity on the surface and the PID gains were optimized to give the higher scan rate compatibly with a reliable scan of the surface. The optimal tip velocity was found to be 20 µm/s. The scan size was fixed at a value of 5µm × 5µm. To obtain a reliable estimation of the size distribution of the nanoparticles, a large number of them were extracted from the images and singly analyzed. The analysis consisted in the estimation of the height of the particle with respect to the background plane and consequently only well isolated nanoparticles were considered. In particular, the height was estimated by evaluating the section of the AFM surface along a line passing through the highest point (the top) of the nanoparticle.”

The surface functional groups of CDs were investigated by Fourier transform infrared (FTIR) on a PerkinElmer Spectrum Two™ IR spectrometer (Waltham, MA, USA). Samples were prepared as pellets of KBr and then dried in vacuum for the analysis.

### 2.3. Optical Characterization

A double beam spectrophotometer (JASCO V-560, Tokjo, Japan) was used to investigate the absorption properties of each fractions of CDs_18.5bar_, CDs_13bar_ and CDs_8bar_ in the range of 200–700 nm. The fluorescence emission properties were detected by an intensified CCD camera (FLIR T1K/T1020, Wilsonville, OR, USA) under laser excitation at 600 nm. The optical analysis were performed on diluted dispersions of CDs in ultrapure water in a 1 cm cuvette. On the basis of the absorption and emission spectra, we selected the fraction of each synthesis with the best emission in the red region under excitation at 600 nm. 

The photothermal effect of the selected fractions of CDs was determined by exciting an aqueous dispersion of CDs (0.2 mL, 0.1 mg mL^−1^) with an 810 nm diode laser source (GBox 15A/B by GIGA Laser, Wuhan, China) with power fitted at 5 W cm^−2^. The temperature was monitored at fixed intervals of laser exposure by a optic fiber (CEM^®^, Charlotte, NC, USA). Equal volume of water as used as control. 

### 2.4. Dynamic-light Scattering (DLS) and ζ-Potential Analyses of CDs

Dynamic light scattering (DLS) measurements were performed on dispersion of CDs_18.5bar_, CDs_13bar_ and CDs_8bar_ (1 mL; 0.5 mg mL^−1^) in both ultrapure water and supplemented DMEM, at 37 °C with a Malvern Zetasizer NanoZS instrument (Malvern Panalytical ltd, Almelo, Netherlands) equipped with a 532 nm laser with a fixed scattering angle of 173°. ζ-Potential analyses were performed using the same conditions reported for the DLS measurements and applying the principle of laser Doppler velocity and phase analysis light scattering (M3-PALS technique) by dispersing samples in the medium and analyzing the electrophoretic mobility of the particles for 2 min at 37 °C under constant electric field. Each sample was analyzed in triplicate and the average ζ-Potential was calculated from the electrophoretic mobility using the Smoluchowski relationship and assuming that K × a >> 1 (where K and a are the Debye-Hückel parameter and particle radius, respectively) [22].

### 2.5. In Vitro Cytocompatibility Assay 

The cytocompatibility of the previously selected fractions of CDs were assessed on human breast cancer cell lines (MDA-MB-231 by Sigma Aldrich, Milan, Italy) by MTS assay (Promega). Cells were seeded on a 96 multiwell plate at a density of 1.5 × 10^4^ cells/well (200 μL) and grown in supplemented Dulbecco’s Minimum Essential Medium (DMEM). After 24 h, the medium was replaced with 200 μL of fresh culture medium containing the CDs at range of concentration from 1 to 0.05 mg mL^−1^. After 48 h, MTS assay was performed replacing the dispersion of CDs in DMEM with 100 μL of fresh medium and 20 μL of MTS solution. After 2 h of incubation at 37 °C and 5% of CO_2_, the absorbance at 492 nm was measured using a microplate reader (Eppendorf PlateReader AF2200, Hamburg, Germany). Untreated cells was used as negative control. In another experimental set was evaluated the NIR-triggered photothermal ablation on MDA-MB-231 of the selected fractions of CDs_18.5bar_, CDs_13bar_ and CDs_8bar_. Cells were seeded in a 96 multiwell as described above. After 48 h of incubation with the dispersions of CDs in DMEM (1–0.05 mg mL^−1^), each well was irradiated with an 810 nm laser for 300 s with power fitted at 5 W cm^−2^. The hyperthermic effect was reported as percentage reduction of the control cells. All culture experiments were performed in triplicate. 

### 2.6. In Vitro Uptake Study on MDA-MB-231 Cell Cultures

The capacity of the various CDs to enter into MDA-MB-231 was evaluated by fluorescence microscopy after 6 h of incubation. Cells were seeded in a 8 well plate at density of 1 × 10^4^ cells/well (200 μL) and growth in DMEM for 24 h. Subsequently, the medium was replaced with equal volume of fresh medium containing CDs (0.25 mg mL^−1^). After 6 h of incubation nuclei were stained with 4′,6-diamidino-2-phenylindole (DAPI). Each well was washed twice with DPBS pH 7.4 and DAPI solution (100 μL) was added. After 10 min of incubation, cells were washed with DPBS (200 μL × 2). Imaged were recorded using an Axio Cam MRm fluorescence microscope (Zeiss, Oberkochen, Germany).

## 3. Results

### 3.1. Preparation and Chemical Characterization of Carbon Nanodots at Different Pressure

**Figure 1:**
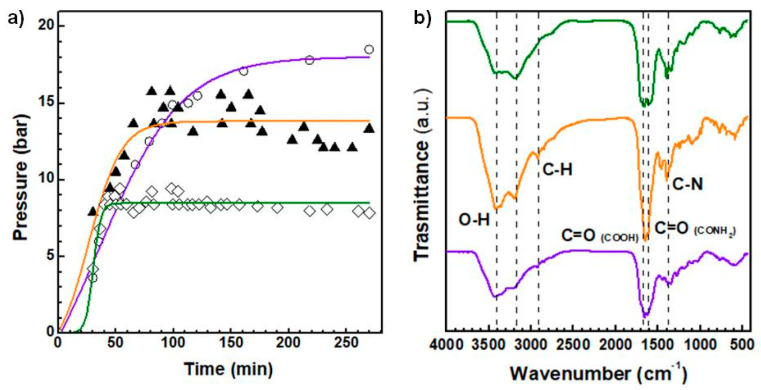
Trend of the pressure registered during solvothermal syntheses for CDs_8bar_ (green line), CDs_13bar_ (orange line) and CDs_18.5bar_ (purple line) (**a**). IR spectra of selected reference fractions of the CDs (**b**): CDs_8 bar_ (green line), CDs_13bar_ (orange line) and CDs_18.5bar_ (purple line).

CDs with different emission spectra were prepared from the solvothermal reaction of urea and citric acid in N-N-dimethylformamide (DMF) by modulating the operating pressure of the reactor. Three pressure were chosen, namely 8, 13 and 18.5 bar, to study the pressure-dependent behavior of the emission spectrum and size distribution of these kind of CDs. Figure 1a shows the trend of the pressure registered during the reactions, suggesting that after 50 min of warm up solvothermal reactions reached the selected pressure to maintain a plateau over time. Only for the higher pressure (18.5 bar) the plateau was registered after 150 min. After purification by size exclusion chromatography (SEC) three main fractions per each reaction were isolated: usually, one emits in the green region of the visible spectrum, another in the orange and the last prevailingly in the red region (data not shown). The reaction yield of the three fractions is strictly correlated with the operating pressure (Table 1). In particular, the yield of CDs emitting in the red region increased by increasing the pressure (4%, 8 bar; 5.7%, 13 bar and 11.6%, 18.5 bar), suggesting that red-emitting CDs can be obtained with good yield at the higher pressure studied. On the contrary, the yield of the green fractions decreased with the operating pressure (from about 14% to 1%, at 8 and 18.5 bar respectively), whereas the yield of the orange fractions had a maximum at 13 bar and decreased at the higher and lower pressures (Table 1). 

With the aim of comparing only the red-emitting CDs obtained in the various synthesis (henceforth named CDs_8bar_, CDs_13bar_ and CDs_18.5bar_), selected for the interest in bioimaging applications, the chemical composition of the surface was established by FTIR analysis reported in Figure 1b. The FTIR spectra of the selected fractions of CDs_18.5bar,_ CDs_13bar_ and CDs_8bar_ show diagnostics bands typical of hydroxyl, carbonyl and amide groups relative to O-H stretching (3436 cm^−1^), C = O stretching (1681 cm^−1^) and amide band (1602 cm^−1^). There are also bands attributable to C-N vibration (1384–3178 cm^−1^) and C-H vibration (2915 cm^−1^) [1,2,23]. In detail, the analysis of the carboxyl/amide band heights highlights that in the CDs_18.5bar_ sample there is a higher amount of carboxylic acid groups on the CDs surface compared to the samples obtained at lower pressure. On the contrary, the CDs_13bar_ and CDs_8bar_ would seem richer in amide groups than carboxyl groups. These data were also confirmed by ζ-Potential measurements reported in Table 2, where it is self-evident that CDs_18.5bar_ have a higher amount of surface negative charge owing to carboxylic acids (−33 mV and about −19 mV for CDs_18.5 bar_ and CDs_13bar_/CDs_8bar_, respectively)

### 3.2. Size Distribution Characterization of the CDs

**Figure 2:**
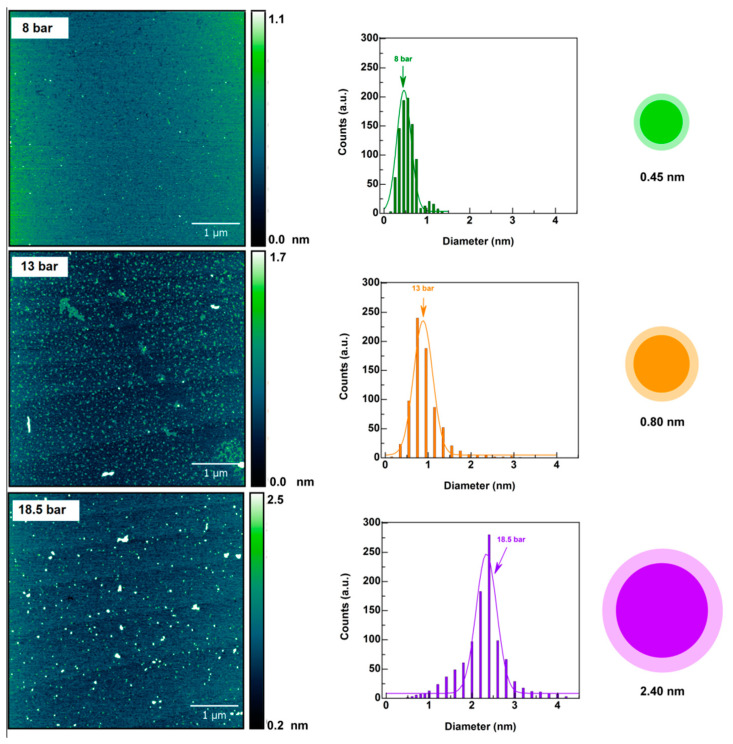
Atomic force microscopy (AFM) micrographs of CDs obtained at increasing pressure (8 → 13→ 18.5 bar) and the relative size distribution diagrams (right) obtained from the heights of the AFM images.

The atomic force microscopy (AFM) was used to evaluate the influence of the operating pressure on the size distribution of the CDs and the involvement of the particle size distribution in emission phenomena by interfacial electron mechanisms. CDs_18.5bar_, CDs_13bar_ and CDs_8bar_ micrographs are reported in Figure 2, together with the size distribution obtained from the particle heights. As can be seen in Figure 2 CDs with narrow size distribution can be obtained after the purification process adopted. The mean diameter varied from 0.45 to 2.4 nm passing from 8 to 18.5 bar, suggesting that tuning the operating pressure during the reaction allows modulating size distribution of the CDs. It might be noticed that non spherical CDs were obtained at low pressure, consisting of a few atoms in height (0.45 nm) extended unidirectionally up to about 1 nm in width. 

### 3.3. Optical Characterization of the CDs

**Figure 3:**
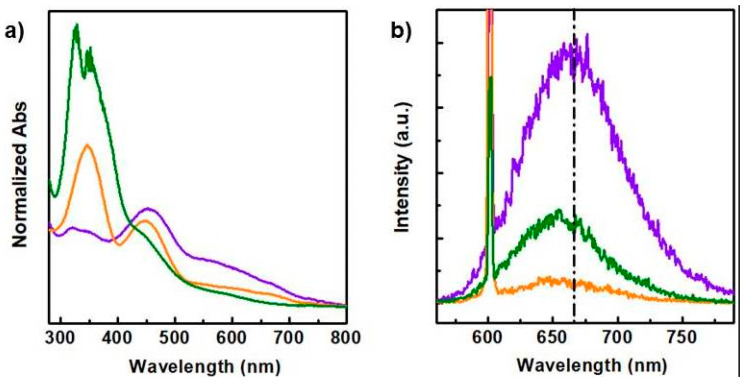
Absorption (**a**) and emission (**b**) spectra of CDs_8bar_ (green line), CDs_13bar_ (orange line) and CDs_18.5bar_ (purple line). λ_ex_ = 600 nm.

To assess the red emission properties of the selected fractions, CDs_18.5bar_, CDs_13bar_ and CDs_8bar_, absorption spectrum and emission spectra were recorded and reported in Figure 3. All CDs are characterized by a complex absorption spectrum consisting of three main bands at about 350, 450 and 600 nm (Figure 3a). These bands are associated to the three main emission bands in the green, orange and red emission region, respectively. It is noteworthy that the relative ratio between these absorption bands significantly changed for the different CDs samples. In particular, the band at 350 nm is particularly remarked for the CDs_8bar_ sample, decreased for the CDs_13 bar_ and hinted for the CDs_18.5bar_ one. Whereas the bands at 450 and 600 nm sharply increased in the CDs_18.5bar_ and CDs_13bar_ spectra, with a predominant band at 600 nm for the CDs_18.5bar_. In order to investigate the emission properties of the CDs in the red region, emission spectra were recorded under excitation wavelength of 600 nm (Figure 3b). It was observed a significant red shift of 10 nm for the CDs_18.5bar_ if compared with parent samples (CDs_8bar_ and CDs_13bar_). The quantum yield (QY%) observed was also higher for the CDs_18.5 bar_ (4.3% vs. 2.4%), implying that the higher operating pressure adopted in the synthesis of CDs (18.5 bar) provided significant improvements in the emission properties of CDs in the red region, desirable for medical applications [10,23]. 

### 3.4. Photothermal Behavior of the CDs

**Figure 4:**
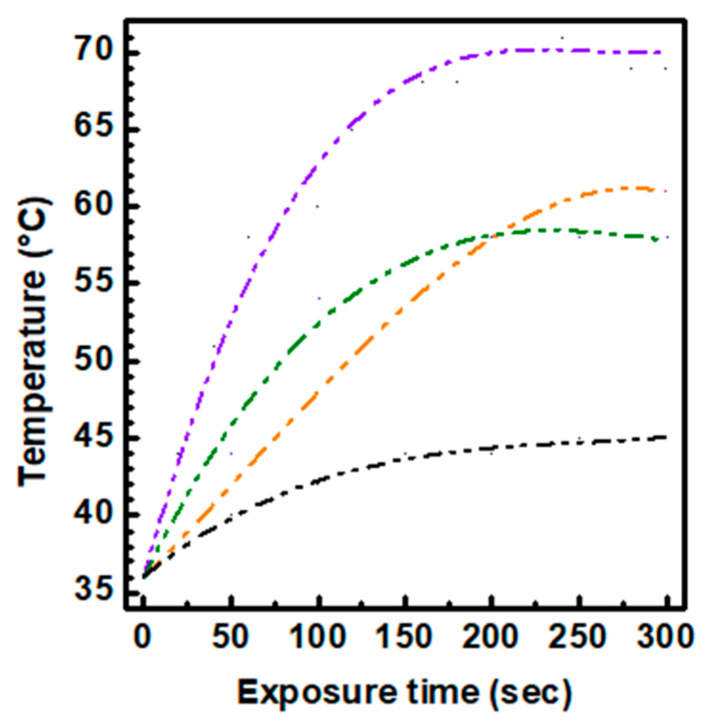
Photothermal kinetics of CDs_8bar_ (green line), CDs_13bar_ (orange line) and CDs_18.5bar_ (purple line) compared with ultrapure water treated under the same conditions (black line). Samples (0.1 mg mL^−1^) were excited with an 810 nm diode laser source with power of 5 W cm^−2^.

To study the photothermal abilities of the selected red-emitting fractions obtained at different operating pressure, CDs_18.5bar_, CDs_13bar_ and CDs_8bar_, each sample (0.2 mL) at a concentration of 0.1 mg mL^−1^ was excited using a diode laser of 810 nm with a power of 5 W cm^−2^ for 300 s and temperature was measured and reported as function of the exposure time (Figure 4). Ultrapure water was used a negative control for comparative purposes. All CDs samples proved capable of increasing the temperature of the medium under NIR exposure up to at least 55 °C, far beyond the photothermal threshold useful in cancer hyperthermia (43 °C). However, as expected the CDs_18.5bar_ sample provided a much more distinguishable photothermal ability, since it allows reaching 79 °C after 300 s exposure. According to the higher absorption property observed in the red wing of the spectrum for the CDs_18.5bar_ sample (Figure 3a), the photothermal conversion of NIR light into heat results strongest for the CDs obtained at the higher pressure, although all CDs sample possess suitable photothermal capabilities useful in cancer therapy. More importantly, all CDs allow tuning NIR photothermal effect so as to generate hyperthermia (43 °C) by easily controlling the exposure time, which could supply the minimum temperature needed to eradicate tumors and avoid damages in healthy tissues [12,24,25]. In particular, under the selected experimental conditions the medium reached 43 °C after barely 25 s of laser exposure for the CDs_18.5bar_ sample, whereas the same temperature was obtained after 40 s and 75 s for the CDs_13 bar_ and CDs_8bar_, respectively.

### 3.5. Cytocompatibility of the CDs and Photothermal-Induced Cancer Cell Death

**Figure 5:**
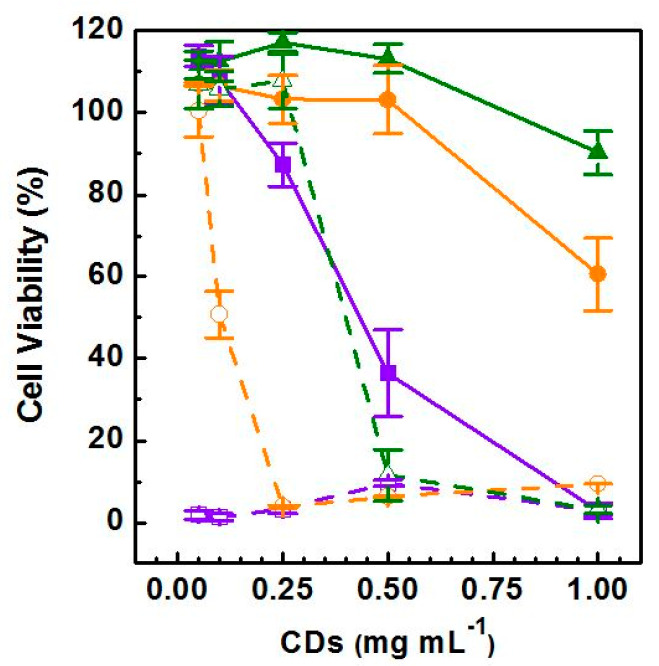
Cytocompatibility on breast cancer cell line (MDA-MB-231) after 48 h of incubation: CDs_18.5bar_ (violet solid line), CDs_13bar_ (orange solid line) and CDs_8bar_ (green solid line). Cytocompatibility after NIR-triggered photothermal treatment (810 nm, power of 5 W cm^−2^): CDs_18.5bar_ (violet dash line), CDs_13bar_ (orange dash line) and CDs_8bar_ (green dash line).

In vitro cytocompatibility of the selected red-emitting fraction of CDs_18.5bar_, CDs_13bar_ and CDs_8bar_ was evaluated on breast cancer cells (MDA-MB-231) after 48 h incubation within a concentration range of 0.05–1 mg mL^−1^. Cell viability is expressed as percentage of cells alive with respect to the untreated control (100% viability). Results are reported in Figure 5. Overall, cell viability followed a dose-dependent trend under the range of concentration considered. In particular, cell viability decreased passing from 0.05 to 1 mg mL^−1^. However, the cytocompatibility profiles of the CDs obtained at different pressure were different. In particular, being cell viability always beyond 90%, CDs_8bar_ display an excellent cytocompatibility over the entire range studied, whilst a lower cytocompatibility was observed for the CDs_13bar_ sample at the higher concentration tested. Surprisingly, the CDs_18.5bar_ sample provoked a significant cell death at concentration higher than 0.25 mg mL^−1^, suggesting that the different nature of the CDs surface observed might play a crucial role in the biological performance of these nanomaterials. 

Considering the controllable high photothermal performance of the red-emitting CDs, as previously demonstrated, we assessed their ability to induce human breast cancer cells death by NIR-triggered photothermal ablation. Therefore, the second experimental set was conducted on MDA-MB-231 after an incubation time of 48 h by irradiating wells with an 810 nm diode laser for 300 s with power fitted at 5 W cm^−2^. The photothermal ablation was measured as a reduction in cell viability after the treatment and results were compared with cells incubated with the CDs without applying the photothermal insult (Figure 5). According with literature data, the 810 nm diode laser treatment alone at suitable power density (0.1–10 W cm^−2^) has a biostimulating effect, which reflects on cell proliferation and thus is considered biocompatible [12,26,27,28]. Whereas, cells incubated with either CDs_18.5bar_, CDs_13bar_ or CDs_8bar_ show a remarkable dose-response reduction in cell viability after laser irradiation, with some qualification. In particular, the photothermal ablation observed for the CDs_18.5bar_ sample was drastic (100% cell death) also at very low dosage (0.05 mg mL^−1^), implying that notwithstanding the lower cytocompatibility showed for these CDs (<0.25 mg mL^−1^) they provide outstanding photothermal-induced anticancer effects within the therapeutic window. In addition, as expected, because of the enhanced photothermal kinetic the photothermal anticancer effect observed for the CDs_13bar_ sample was much more potent than that displayed by the CDs_8bar_ ones. In fact, CDs_13bar_ induce 50% of cell death (IC_50_) at a concentration of 0.10 mg mL^−1^ and almost the complete cell death at 0.25 mg mL^−1^, two times more potent than that showed by CDs_8 bar_ (IC_50_ > 0.48 mg mL^−1^). 

### 3.6. Evaluation of Serum Protein Interactions by Dynamic Light Scattering and z-Potential Measurements

When nanoparticles with high specific surface and high surface charge are in biological fluids, they can interact with proteins to form a protein shell with various conformations named protein corona, which can affect their physicochemical properties and the ability to enter cells [19,20,21,29,30,31]. Thus the interaction of the selected red fractions of CDs_18.5bar_, CDs_13bar_ and CDs_8bar_ with DMEM serum protein was established exploring the aggregation of them after incubation at 37 °C by DLS measurements. As shown in Table 2, all CDs samples interact with DMEM proteins giving rise to bigger nanoparticles within the range of 120–270 nm in diameter. In particular, it seems that CDs with the higher amount of surface carboxylic functions, namely CDs_18.5bar_ and CDs_13bar_, yield to stronger interactions with DMEM affording to nanoparticles of roughly 210 and 270 nm in diameter, respectively. The involvement of electrostatic interactions on the CDs surface in the protein corona adsorption process was also established by ζ-Potential measurements (Table 2). For all the CDs tested the ζ-Potential results negative in pure water and decreased up to about −6 mV once the nanoparticles were dispersed in DMEM. The reduction of the ζ-Potential provoked a time-dependent flocculation of the CDs into microparticles. 

### 3.7. In Vitro Uptake Study on the MDA-MB-231 Cell Line

**Figure 6:**
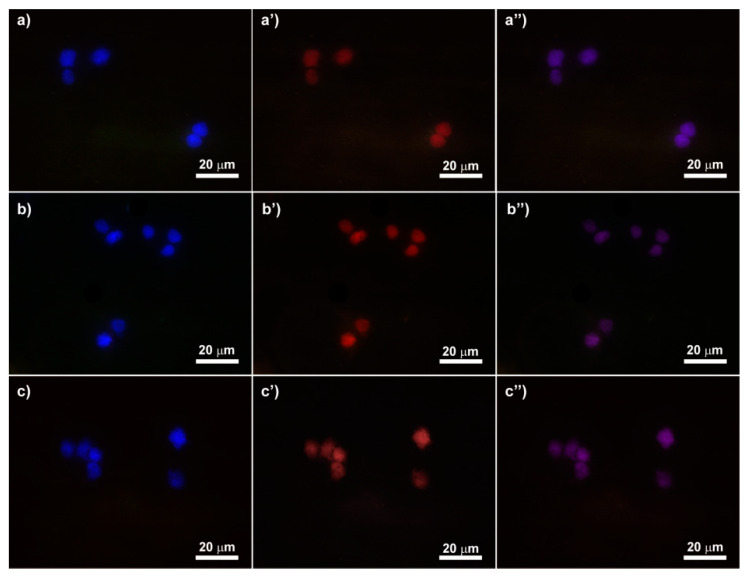
Fluorescence micrographs of the selected fraction of CDs_18.5bar_ (**a**–**a″**), CDs_13bar_ (**b**–**b″**) and CDs_8bar_ (**c**–**c″**) after 6 h of incubation on MDA-MB-231. Magnification 40×: nucel (blue; **a**, **b** and **c**), CDs (red; **a,′ b′** and **c′**) and merge (**a″**, **b″** and **c″**).

To assess the potential role of the selected red-emitting fractions of CDs_18.5bar_, CDs_13bar_ and CDs_8bar_ in intracellular delivery and single cell imaging applications, MDA-MB-231 cells were incubated with an equivalent amount of either CDs_18.5bar_, CDs_13bar_ or CDs_8bar_ for 6 h and cell uptake was evaluated using fluorescence microscopy tracking the red self-fluorescence of CDs and blue fluorescence of nuclei marked with a DAPI solution (Figure 6). Figure 6 shows a certain ability of all CDs samples to enter cancer cells under physiological conditions in DMEM, since red fluorescence co-localized inside nuclei can be observed for all CDs after 6 h incubation (Figure 6a–c). However, as expected, cell cultures were highly contaminated by aggregates visible before washing up wells with DPBS using the Texas red channel (data not shown) corroborating the tendency of CDs to provide significant aggregation in DMEM. The presence of microparticles (d > 200 nm) explains quite well why all CDs moderately enter MDA-MB-231 cells under physiological conditions. 

## 4. Discussion

In our previous works, we have demonstrated that red-emitting carbon nanodots (CDs) with very narrow size distribution and excellent NIR-triggered photothermal capabilities can be obtained by the solvothermal reaction of urea and citric acid in DMF in sealed autoclave [1,3]. We showed that these CDs are effective platforms for delivering anticancer drugs and simultaneously allowing the selective photothermal ablation of tumors by applying an external NIR laser source at low power density. Many research groups have debated on the possibility of tuning photoluminescence of CDs as function of monomer ratios, reaction temperature, solvent composition and doping agents, in order to obtain CDs with optical properties suitable for optoelectronics, catalysis and biomedical applications [32,33,34]. However, despite the fact that pressure is an undisputed key thermodynamic factor which drive the formation of crystals during solvothermal reactions, there is no information on the role of the operating pressure in influencing the formation of nitrogen-rich carbon nanodots and their optical, morphological and physicochemical properties [35]. In this work, we investigated the effect of operating pressure on the optical, NIR photothermal conversion and size distribution of nitrogen-rich CDs obtained by the well-established bottom-up decomposition of citric acid and urea in DMF. CDs were synthesized in condition of nitrogen doping, since it seems to be crucial to have a good emission efficiency. We tested three very different pressures, namely 8, 13 and 18.5 bar by simply tuning a manual valve applied on the cap of the autoclave. Micrometric tuning of the valve allows reaching the selected operating pressure within 45 min, thus keeping the operating pressure for about 2 h (Figure 1a). These conditions are suitable to provide sufficient time for the formation of ordered crystals with the typical structure of nitrogen-rich CDs reported elsewhere [1]. Under the selected conditions all the CDs obtained consisted of a mixture of amorphous carbonaceous materials and heterogeneous green, orange and red emitting CDs but with different composition. The use of size exclusion chromatography (SEC) allowed to isolate different fractions with peculiar size, surface and optical properties. In detail, CDs were purified by a strategic combination of two sephadex stationary phases packed with increasing cut-off from G15 to G25, in order to eliminate by-products and smallest materials. Then, a first analysis of the optical properties allows to select fractions of each sample of CDs with the best red fluorescence. Red fluorescence was selected because of red-to-NIR fluorescence is required in fluorescence imaging applications to obtain high resolution image within the biologically transparent window. However, the yield of the red fraction was strongly affected by the operating pressure and increased with the pressure from 4% to 12% *w*/*w* (Table 1). Overall, CDs obtained at 8 bar were prevailingly green as the ones obtained at 13 bar were prevailingly orange and finally the CDs obtained at 18.5 bar, the highest pressure registered for this kind of reaction, resulted prevailingly red. 

Red fractions of each reaction were further characterized to attain the surface chemical structure by FTIR analysis. The surface functional groups present on the CDs surface were typically amide, hydroxyl and carboxyl groups but in different ratios. In particular, the amount of carboxyl groups increased by increasing the operating pressure, whereas the amide groups followed an opposite trend (Figure 1b and Table 2). It is well known that surface composition is a crucial factor for nanomaterials, since affects many parameters which take part a role at the biomaterial/tissue interface such as cell uptake, nano-toxicity, protein absorption, biodistribution and immune system activation [36,37]. Thus, the operating pressure could be a key parameter to modulate the yield of red-emitting CDs and their surface composition as well as the biological fate of these class of CDs.

Another parameter that influence interactions between biomaterials and tissues is the size distribution of nanoparticles [36,37]. Even if CDs are enough small (<5 nm) to be eliminated by normal renal clearance, interactions with blood and tissues also depends on the size distribution of nanoparticles and processes that allow tuning this parameter could be of help in the design of effective nanomedicines with negligible side toxic effects [38]. Here it is showed that the operating pressure provides a simple way to obtain highly homogeneous CDs with size distribution proportional to the pressure adopted (Figure 2). It is noteworthy that high pressure is required to provide spherical CDs and complete multidirectional crystal growth, while at lower pressure (8 bar) crystal growing results incomplete giving rise to CDs of a few Angstroms in height. The mechanism of this phenomenon is associated with directional stress on solubility of compounds produced during the dehydration and carbonization of the starting monomers. In fact, under a compressive stress the solubility of the stressed faces is higher and crystal growth of the stressed faces depends on the degree of supersaturation of the liquid [39]. 

Previews publications report that CDs with red/NIR fluorescence can be obtained by doping nanoparticles with sulphur or compounds able to be incorporated into the crystals during the growing processes. However using this strategy the control of the chemical composition and physicochemical properties of CDs is biased and results arduous to produce standard nanoparticles suitable for biomedical use. Here, we attempt to examine a simple procedure to obtain nitrogen-rich CDs with a significant red shift by controlling crucial synthetic parameters such as the pressure. Being photoluminescence of CDs tuned by changing their surface properties and size distribution, the influence of pressure on size and functional groups density above observed induced us to study the effect on their emission properties (Figure 3) [40]. It might be noticed that all samples are characterized by three main absorption bands at 350, 450 and 600 nm, which correspond to the emission bands from green to red, respectively (Figure 3). However, the relative intensity of these bands significantly changed for the CDs obtained at different pressure. In particular, the absorption band at 350 nm decreased by increasing the operating pressure, while the band at 600 nm, responsible for the red emission, increased with the operating pressure adopted during the synthesis. In the emission spectra recorded using an excitation wavelength of 600 nm it was found a desirable red shift at increasing pressure, especially for the CDs_18.5bar_ sample, confirming that to date the operating pressure is an underestimate parameter in solvothermal synthesis of CDs which provide a versatile and fine control of the physicochemical properties of CDs. Besides, the enhanced absorption tail at 600–800 nm revealed for the CDs_18.5bar_ (Figure 3a) suggested that CDs with efficient NIR photothermal conversion can be obtained at higher pressure (Figure 4). The photothermal kinetics under NIR laser excitation confirm that increasing pressures ensure, together with optimal fluorescence in the transparent window, a higher photothermal performance in the NIR region, needed for deep tissue image-guided phototherapy.

With the aim of understanding the anticancer potential of the selected red-emitting CDs_18.5bar_, CDs_13bar_ and CDs_8bar_, in vitro cytocompatibility MTS assay was performed on MDA-MB-231 cells, used as a gold standard of triple negative breast cancer cell line. Although the cytocompatibility profile of the three CDs appeared very different on the basis of their physicochemical properties, all CDs had a good potential therapeutic window which provided on demand efficient photothermal ablation of tumor cells only after the NIR laser exposure for 300 seconds at suitable power density (Figure 5). As a rule, CDs_8bar_ with lower amount of carboxylic acid and lower ζ-Potential (≈−19 mV) are much more cytocompatible, while cell viability tends to decrease at the higher dosage tested (0.5–1 mg mL^−1^) for the CDs obtained at higher pressure (Figure 1, Table 2). This behavior can be explained considering that CDs are prone to provoke electrostatic adsorption of serum proteins of the medium on their surface to give rise to nanoparticle of about 270 nm in diameter and quasi null ζ-Potential (≈−6 mV) (Table 2). As consequence, the amount of corona protein on the surface is proportional to the size of the nanoparticles (CDs_18.5 bar_ are five times bigger than CDs_8 bar_, Figure 2) and, in particular, should be more abundant in larger nanoparticles. This could significantly change the biological behavior of nanoparticles suggesting that corona protein might be involved in cytotoxicity mechanisms of such nanoparticles [18].

Although CDs_8bar_ show the best profile of cytocompatibility, they need higher concentration to induce cancer cells death by photothermal ablation (IC_50_ = 0.45 mg mL^−1^) if compared with the parent CDs (CDs_13bar_, IC_50_ = 0.125 mg mL^−1^; CDs_18.5bar_, IC_50_ < 0.05 mg mL^−1^). Similar to other tested nano-heaters, the treatment of MDA-MB-231 with in itself harmless dosage of NIR diode laser (300 s, 5 W cm^−2^) provoked activation of CDs within the therapeutic window and the local release of heat so as to provoke a selective cell death [41,42,43,44]. It is noteworthy that, according to literature, the exposure of cells to the laser alone has biostimulating properties and usually yield to an increase in cell viability [12,26,45]. Besides, CDs_18.5 bar_ was able to provoke 100% of cell death under NIR laser treatment at very low dosage (<50 μg mL^−1^), implying absolute eradication of cancer cells which help to avoid recidivisms. Indeed, it is reported that inhibition of apoptosis in cancer cells after chemical or physical stress plays a critical role in cancer cell survival and tumor development [46]. Thus the operating pressure allow tuning both physicochemical and biological properties of CDs, providing a key synthetic parameter to design new nanomaterials adaptable according to medical needs.

Besides, we have focused our attention on understanding how to modulate the features of CDs to combine excellent biological, photothermal and fluorescence imaging properties useful in image-guided cancer phototherapy. Increasing the operating pressure we observed improved QY in the red region (from 2.4% to 4.3%) that provides self-tracking capabilities in fluorescence imaging applications such as single cell imagining using fluorescence microscopy. This observation was also confirmed by the uptake study on MDA-MB-231 cells, as, despite the aggregation observed owing to the formation of corona protein, after 6 h of incubation cell uptake was clearly visible at the nuclear region since a red fluorescence attributable to CDs co-localized with the blue one of DAPI bounded inside nuclei (Figure 6). Furthermore, for the CDs_18.5bar_ sample red fluorescence was much more marked, probably related to a higher QY in the red region (QY = 4.3%). Thus, this is a good evidence that CDs have a good potential in imaging-guided applications for precision cancer therapy.

Overall, our results indicate that pressure conditions impinge on the formation of nitrogen-rich carbon nanoparticles under solvothermal conditions and influence their characteristics. Pressure can be tuned to impart desired optical, size distribution, surface functionalization and anticancer properties to CDs in a facile and scalable way. Therefore, pressure as well as temperature and the strategic combination of solvents and reagents can be exploited to obtain CDs with good red/NIR fluorescence and photothermal properties for anticancer theranostic applications. However, we have highlighted a strong tendency of the obtained CDs to interact with serum proteins forming aggregates with a diameter of approximately 130–280 nm, that limiting their in vivo use. A strategic surface engineering of these CDs need to be considered to limit the adsorption of corona proteins and also to increase the average surface diameter, avoiding a rapid renal clearance and improving their therapeutic efficacy in vivo.

## 5. Conclusions

In this work, we highlighted an interesting strategy to manage the bottom up synthesis of carbon nanodots so as to tune fluorescence, size distribution, surface functional groups, photothermal behavior and their biological performance. We recognized the operating pressure as a key thermodynamic parameter to design CDs with defined size distribution, optical and biological properties. Experiments conclusively point out that the formation of CDs in solvothermal decomposition of citric acid and urea involves pressure-dependent crystal growth mechanisms which significantly impinge on all physicochemical and biological characteristics of CDs, crucial parameters to develop theranostic tools with tailor made properties useful in imaging-guided photothermal applications.

The fluorescence displays a red shift related to the pressure adopted (increases with pressure) and the yield of red-emitting carbon nanodots increased at higher pressure, implying that the emission band can be tuned by simply changing pressure. Besides, CDs obtained at the higher pressure show a huge absorption tail in the NIR region, responsible for the good NIR photothermal conversion observed. AFM data display that also the size distribution increased at higher pressure, passing from 0.45 to 2.4 nm. Indeed, incomplete crystal growth was observed at 8 bar, the lower pressure tested.

Preliminary biological studies on breast cancer cells allowed establishing that CDs proved capable of acting as nanoheaters useful to induce on demand cell death at cytocompatible dosage, combined with self-tracking abilities in fluorescence imaging. The potency of the photothermal treatment was proportional to the operating pressure adopted. However, we found that further surface engineering of CDs exploiting their surface functional groups is required to avoid adsorption of protein corona, which strongly influence the interaction of CDs with living cells.

## Figures and Tables

**Table 1:** Reaction yield of the carbon nanodots (CDs) obtained at different operating pressure.

Sample	Yield_Green_^1^ (%)	Yield_Orange_^1^ (%)	Yield_Red_^1^ (%)
CDs_8bar_	14.3	6.4	4.0
CDs_13bar_	5.4	8.1	5.7
CDs_18.5bar_	0.9	4.1	11.6

^1^ Calculated on a weight basis considering the weight of starting monomers completely dehydrated.

**Table 2:** Z-Average, PDI and ζ-Potential of red CDs_18.5bar_, CDs_13bar_ and CDs_8bar_ (0.25 mg mL^−1^).

Sample	Z-Average ^1^ (nm)	PDI^1^	Z-Average ^2^ (nm)	PDI ^2^	ζ-Potential ^1^ (mV)	ζ-Potential ^2^ (mV)
CDs_8bar_	1.9	0.06	128.3	0.253	−18.4 ± 6.5	−5.5 ± 1.2
CDs_13bar_	2.4	0.1	267.5	0.498	−19.6 ± 8.6	−6.0 ± 1.1
CDs_18.5bar_	5.3	0.02	210.8	0.373	−33.6 ± 5.9	−6.2 ± 1.7
DMEM	-	-	91.8	0.219	-	−10.1 ± 8.4

^1^ Data obtained by dynamic light scattering analysis in ultrapure water at 37 °C. ^2^ Data obtained by dynamic light scattering analysis in supplemented DMEM at 37 °C.

## References

[1] Scialabba C., Sciortino A., Messina F., Buscarino G., Cannas M., Roscigno G., Condorelli G., Cavallaro G., Giammona G., Mauro N. (2019). Highly Homogeneous Biotinylated Carbon Nanodots: Red-Emitting Nanoheaters as Theranostic Agents toward Precision Cancer Medicine. ACS Appl. Mater. Interfaces.

[2] Sciortino A., Mauro N., Buscarino G., Sciortino L., Popescu R., Schneider R., Giammona G., Gerthsen D., Cannas M., Messina F. (2018). β-C_3_N_4_ Nanocrystals: Carbon Dots with Extraordinary Morphological, Structural and Optical Homogeneity. Chem. Mater..

[3] Gazzetto M., Sciortino A., Nazari M., Rohwer E., Giammona G., Mauro N., Feurer T., Messina F., Cannizzo A. (2020). Photocycle of Excitons in Nitrogen-Rich Carbon Nanodots: Implications for Photocatalysis and Photovoltaics. ACS Appl. Nano Mater..

[4] Bhattacharyya S., Ehrat F., Urban P., Teves R., Wyrwich R., Döblinger M., Feldmann J., Urban A.S., Stolarczyk J.K. (2017). Effect of nitrogen atom positioning on the trade-off between emissive and photocatalytic properties of carbon dots. Nat. Commun..

[5] Lim S.Y., Shen W., Gao Z. (2015). Carbon quantum dots and their applications. Chem. Soc. Rev..

[6] Liang Y., Liu Y., Li S., Lu B., Liu C., Yang H., Ren X., Hou Y. (2019). Hydrothermal growth of nitrogen-rich carbon dots as a precise multifunctional probe for both Fe3+ detection and cellular bio-imaging. Opt. Mater..

[7] Al-Hashimi B., Rahman H.S., Omer K.M. (2020). Highly luminescent and biocompatible P and N Co-doped passivated carbon nanodots for the sensitive and selective determination of rifampicin using the inner filter effect. Materials.

[8] Mishra V., Patil A., Thakur S., Kesharwani P. (2018). Carbon dots: Emerging theranostic nanoarchitectures. Drug Discov. Today.

[9] Robinson J.T., Tabakman S.M., Liang Y., Wang H., Sanchez Casalongue H., Vinh D., Dai H. (2011). Ultrasmall reduced graphene oxide with high near-infrared absorbance for photothermal therapy. J. Am. Chem. Soc..

[10] Zhou F., Xing D., Ou Z., Wu B., Resasco D.E., Chen W.R. (2009). Cancer photothermal therapy in the near-infrared region by using single-walled carbon nanotubes. J. Biomed. Opt..

[11] Ðorđević L., Arcudi F., Prato M. (2019). Preparation, functionalization and characterization of engineered carbon nanodots. Nat. Protoc..

[12] Mauro N., Scialabba C., Cavallaro G., Licciardi M., Giammona G. (2015). Biotin-containing reduced graphene oxide-based nanosystem as a multieffect anticancer agent: Combining hyperthermia with targeted chemotherapy. Biomacromolecules.

[13] Wang Q., Liu X., Zhang L., Lv Y. (2012). Microwave-assisted synthesis of carbon nanodots through an eggshell membrane and their fluorescent application. Analyst.

[14] Kappe C.O. (2004). Controlled microwave heating in modern organic synthesis. Angew. Chem. Int. Ed..

[15] Demazeau G. (2008). Solvothermal processes: New trends in materials chemistry. Proc. J. Phys. Conf. Ser..

[16] Miao X., Yan X., Qu D., Li D., Tao F.F., Sun Z. (2017). Red Emissive Sulfur, Nitrogen Codoped Carbon Dots and Their Application in Ion Detection and Theraonostics. ACS Appl. Mater. Interfaces.

[17] Smith A.M., Mancini M.C., Nie S. (2009). Bioimaging: Second window for in vivo imaging. Nat. Nanotechnol..

[18] Khanal D., Lei Q., Pinget G., Cheong D.A., Gautam A., Yusoff R., Su B., Yamaguchi S., Kondyurin A., Knowles J.C. (2020). The protein corona determines the cytotoxicity of nanodiamonds: Implications of corona formation and its remodelling on nanodiamond applications in biomedical imaging and drug delivery. Nanoscale Adv..

[19] Li Y., Lee J.S. (2020). Insights into characterization methods and biomedical applications of nanoparticle-protein corona. Materials.

[20] Chen D., Ganesh S., Wang W., Amiji M. (2019). The role of surface chemistry in serum protein corona-mediated cellular delivery and gene silencing with lipid nanoparticles. Nanoscale.

[21] Czarnecka J., Wisniewski M., Forbot N., Bolibok P., Terzyk A.P., Roszek K. (2020). Cytotoxic or not? Disclosing the toxic nature of carbonaceous nanomaterials through nano-bio interactions. Materials.

[22] Mauro N., Schillaci D., Varvarà P., Cusimano M.G., Geraci D.M., Giuffrè M., Cavallaro G., Maida C., Giammona G. (2018). Branched High Molecular Weight Glycopolypeptide With Broad-Spectrum Antimicrobial Activity for the Treatment of Biofilm Related Infections. ACS Appl. Mater. Interfaces.

[23] Huo F., Liang W., Tang Y., Zhang W., Liu X., Pei D., Wang H., Jia W., Jia P., Yang F. (2019). Full-color carbon dots with multiple red-emission tuning: On/off sensors, in vitro and in vivo multicolor bioimaging. J. Mater. Sci..

[24] Ghosh S., Dutta S., Gomes E., Carroll D., D’Agostino R., Olson J., Guthold M., Gmeiner W.H. (2009). Increased heating efficiency and selective thermal ablation of malignant tissue with DNA-encased multiwalled carbon nanotubes. ACS Nano.

[25] Sarkar S., Levi-Polyachenko N. (2020). Conjugated polymer nano-systems for hyperthermia, imaging and drug delivery. Adv. Drug Deliv. Rev..

[26] Sroka R., Schaffer M., Fuchs C., Pongratz T., Schrader-Reichard U., Busch M., Schaffer P.M., Dühmke E., Baumgartner R. (1999). Effects on the mitosis of normal and tumor cells induced by light treatment of different wavelengths. Lasers Surg. Med..

[27] Mauro N., Scialabba C., Pitarresi G., Giammona G. (2017). Enhanced adhesion and in situ photothermal ablation of cancer cells in surface-functionalized electrospun microfiber scaffold with graphene oxide. Int. J. Pharm..

[28] Mauro N., Scialabba C., Agnello S., Cavallaro G., Giammona G. (2020). Folic acid-functionalized graphene oxide nanosheets via plasma etching as a platform to combine NIR anticancer phototherapy and targeted drug delivery. Mater. Sci. Eng. C.

[29] Tiwari A., Bhatia P., Randhawa J.K. (2020). Systematic spectroscopic investigation of structural changes and corona formation of bovine serum albumin over magneto-fluorescent nanoparticles. Dalton Trans..

[30] Maity A., Pal U., Chakraborty B., Sengupta C., Sau A., Chakraborty S., Basu S. (2019). Preferential photochemical interaction of Ru (III) doped carbon nano dots with bovine serum albumin over human serum albumin. Int. J. Biol. Macromol..

[31] Song Y., Wang H., Zhang L., Lai B., Liu K., Tan M. (2020). Protein corona formation of human serum albumin with carbon quantum dots from roast salmon. Food Funct..

[32] Tian Z., Zhang X., Li D., Zhou D., Jing P., Shen D., Qu S., Zboril R., Rogach A.L. (2017). Full-Color Inorganic Carbon Dot Phosphors for White-Light-Emitting Diodes. Adv. Opt. Mater..

[33] Baker S.N., Baker G.A. (2010). Luminescent carbon nanodots: Emergent nanolights. Angew. Chem. Int. Ed..

[34] Ghosal K., Ghosh A. (2019). Carbon dots: The next generation platform for biomedical applications. Mater. Sci. Eng. C.

[35] Feng S.H., Li G.H. (2017). Hydrothermal and Solvothermal Syntheses. Modern Inorganic Synthetic Chemistry.

[36] Aggarwal P., Hall J.B., McLeland C.B., Dobrovolskaia M.A., McNeil S.E. (2009). Nanoparticle interaction with plasma proteins as it relates to particle biodistribution, biocompatibility and therapeutic efficacy. Adv. Drug Deliv. Rev..

[37] Petros R.A., Desimone J.M. (2010). Strategies in the design of nanoparticles for therapeutic applications. Nat. Rev. Drug Discov..

[38] Huang X., Zhang F., Zhu L., Choi K.Y., Guo N., Guo J., Tackett K., Anilkumar P., Liu G., Quan Q. (2013). Effect of injection routes on the biodistribution, clearance and tumor uptake of carbon dots. ACS Nano.

[39] Dawson A., Allan D.R., Belmonte S.A., Clark S.J., David W.I.F., McGregor P.A., Parsons S., Pulham C.R., Sawyer L. (2005). Effect of high pressure on the crystal structures of polymorphs of glycine. Cryst. Growth Des..

[40] Bao L., Liu C., Zhang Z.L., Pang D.W. (2015). Photoluminescence-tunable carbon nanodots: Surface-state energy-gap tuning. Adv. Mater..

[41] Nam J., Son S., Ochyl L.J., Kuai R., Schwendeman A., Moon J.J. (2018). Chemo-photothermal therapy combination elicits anti-tumor immunity against advanced metastatic cancer. Nat. Commun..

[42] Zheng M., Zhao P., Luo Z., Gong P., Zheng C., Zhang P., Yue C., Gao D., Ma Y., Cai L. (2014). Robust ICG theranostic nanoparticles for folate targeted cancer imaging and highly effective photothermal therapy. Proc. ACS Appl. Mater. Interfaces.

[43] Chen Q., Wen J., Li H., Xu Y., Liu F., Sun S. (2016). Recent advances in different modal imaging-guided photothermal therapy. Biomaterials.

[44] Zhang P., Hu C., Ran W., Meng J., Yin Q., Li Y. (2016). Recent progress in light-triggered nanotheranostics for cancer treatment. Theranostics.

[45] Wang Y., Huang Y.Y., Wang Y., Lyu P., Hamblin M.R. (2017). Red (660 nm) or near-infrared (810 nm) photobiomodulation stimulates, while blue (415 nm), green (540 nm) light inhibits proliferation in human adipose-derived stem cells. Sci. Rep..

[46] Fernald K., Kurokawa M. (2013). Evading apoptosis in cancer. Trends Cell Biol..

